# Limited English Proficiency and Sepsis Mortality by Race and Ethnicity

**DOI:** 10.1001/jamanetworkopen.2023.50373

**Published:** 2024-01-04

**Authors:** Neha P. Limaye, Wilfredo R. Matias, Hallie Rozansky, Bridget A. Neville, Allison Vise, Dustin S. McEvoy, Sayon Dutta, Esteban Gershanik

**Affiliations:** 1 Department of Medicine, Mount Sinai Hospital, New York, New York; 2 Department of Pediatrics, Mount Sinai Hospital, New York, New York; 3 Arnhold Institute for Global Health, Icahn School of Medicine, New York, New York; 4 Division of Infectious Diseases, Massachusetts General Hospital, Boston; 5 Division of Infectious Diseases, Brigham and Women’s Hospital, Boston, Massachusetts; 6 Center for Global Health, Massachusetts General Hospital, Boston; 7 Section of General Internal Medicine, Boston Medical Center, Boston, Massachusetts; 8 Chobanian & Avedisian School of Medicine, Boston University, Boston, Massachusetts; 9 Grayken Center for Addiction, Boston Medical Center, Boston, Massachusetts; 10 Division of General Internal Medicine and Primary Care, Brigham and Women’s Hospital, Boston, Massachusetts; 11 Department of Medicine, Brigham and Women’s Hospital, Boston, Massachusetts; 12 Division of Health Sciences and Technology, Harvard Medical School, Boston, Massachusetts; 13 Mass General Brigham Digital, Boston, Massachusetts; 14 Department of Emergency Medicine, Massachusetts General Hospital, Boston; 15 Deparment of Medicine, Harvard Medical School, Boston, Massachusetts

## Abstract

**Importance:**

Patients with limited English proficiency (LEP) face multiple barriers and are at risk for worse health outcomes compared with patients with English proficiency (EP). In sepsis, a major cause of mortality in the US, the association of LEP with health outcomes is not widely explored.

**Objective:**

To assess the association between LEP and inpatient mortality among patients with sepsis and test the hypothesis that LEP would be associated with higher mortality rates.

**Design, Setting, and Participants:**

This retrospective cohort study of hospitalized patients with sepsis included those who met the Centers for Disease Control and Prevention’s sepsis criteria, received antibiotics within 24 hours, and were admitted through the emergency department. Data were collected from the electronic medical records of a large New England tertiary care center from January 1, 2016, to December 31, 2019. Data were analyzed from January 8, 2021, to March 2, 2023.

**Exposures:**

Limited English proficiency, gathered via self-reported language preference in electronic medical records.

**Main Outcomes and Measures:**

The primary outcome was inpatient mortality. The analysis used multivariable generalized estimating equation models with propensity score adjustment and analysis of covariance to analyze the association between LEP and inpatient mortality due to sepsis.

**Results:**

A total of 2709 patients met the inclusion criteria, with a mean (SD) age of 65.0 (16.2) years; 1523 (56.2%) were men and 327 (12.1%) had LEP. Nine patients (0.3%) were American Indian or Alaska Native, 101 (3.7%) were Asian, 314 (11.6%) were Black, 226 (8.3%) were Hispanic, 38 (1.4%) were Native Hawaiian or Other Pacific Islander or of other race or ethnicity, 1968 (72.6%) were White, and 6 (0.2%) were multiracial. Unadjusted mortality included 466 of 2382 patients with EP (19.6%) and 69 of 327 with LEP (21.1%). No significant difference was found in mortality odds for the LEP compared with EP groups (odds ratio [OR], 1.12 [95% CI, 0.88-1.42]). When stratified by race and ethnicity, odds of inpatient mortality for patients with LEP were significantly higher among the non-Hispanic White subgroup (OR, 1.76 [95% CI, 1.41-2.21]). This significant difference was also present in adjusted analyses (adjusted OR, 1.56 [95% CI, 1.02-2.39]). No significant differences were found in inpatient mortality between LEP and EP in the racial and ethnic minority subgroup (OR, 0.99 [95% CI, 0.63-1.58]; adjusted OR, 0.91 [95% CI, 0.56-1.48]).

**Conclusions and Relevance:**

In a large diverse academic medical center, LEP had no significant association overall with sepsis mortality. In a subgroup analysis, LEP was associated with increased mortality among individuals identifying as non-Hispanic White. This finding highlights a potential language-based inequity in sepsis care. Further studies are needed to understand drivers of this inequity, how it may manifest in other diverse health systems, and to inform equitable care models for patients with LEP.

## Key Points

**Question:**

Is limited English proficiency (LEP) associated with higher rates of inpatient mortality for patients with sepsis?

**Findings:**

In a cohort study of 2709 patients hospitalized with sepsis from 2016 to 2019 at an urban tertiary care center, there was no significant overall difference in inpatient mortality for patients with LEP compared with those with English proficiency. In a subgroup analysis by race and ethnicity, there was significantly higher mortality for non-Hispanic or non-Latinx White patients with LEP when compared with their counterparts with English proficiency.

**Meaning:**

These findings suggest a language-based inequity in outcomes of patients with sepsis that requires further research and intervention.

## Teaser

This cohort study evaluates the association between limited English proficiency and inpatient mortality due to sepsis among hospitalized patients.

## Introduction

Sepsis is the leading cause of death among hospitalized patients, contributing to more than 270 000 deaths annually in the US.^1,2^ Sepsis affects approximately 6% of hospital admissions and is the highest cost condition in US hospitalizations.^1,2,3^ Several initiatives have targeted improving sepsis care, from the 2002 Surviving Sepsis Campaign aimed at standardizing care and reducing mortality, to the 2015 Centers for Medicare & Medicaid Services Severe Sepsis and Septic Shock Management Bundle (SEP-1) quality measure focused on improving early identification and treatment.^4,5^ These initiatives integrated the Institute of Medicine’s quality improvement pillars of safety, effectiveness, efficiency, and timeliness, but none required reporting on the pillars of equity and patient-centeredness, despite long-standing, known inequities in our health care delivery system.^6,7^

Prior to SEP-1 quality measure development, multiple studies demonstrated inequities in outcomes of patients with sepsis by age, sex, race, and ethnicity.^8,9,10,11^ However, despite a population of nearly 25 million people in the US with limited English proficiency (LEP) who historically experience worse care outcomes, increased lengths of stay, and higher readmission rates,^12,13,14,15,16,17,18^ only 1 academic center study^18^ has evaluated the association of LEP with sepsis outcomes. That study found that LEP was associated with increased mortality across most racial groups among patients hospitalized with sepsis, concluding that “further exploration into the causal nature of this association is required.”^18^

Limited English proficiency status may lead to worse sepsis outcomes due to communication barriers, lower-quality care, potential biases, and other factors.^19,20,21,22^ We aimed to evaluate the association between LEP and inpatient mortality due to sepsis at a hospital representing different languages, races and ethnicities, and geography than the original study. We hypothesized that inpatient mortality due to sepsis would be higher among patients with LEP. Our examination of this association in a new population, with new adjustments for socioeconomic status and illness severity, helps further characterize a potential health inequity affecting over 1 million patients annually and may offer opportunities for focused interventions.^2^

## Methods

The Mass General Brigham Human Research Committee Institutional Review Board reviewed the protocol for this cohort study and waived the need for ethics approval and informed consent for collection, analysis, and publication of these retrospectively obtained data. This study followed the Strengthening the Reporting of Observational Studies in Epidemiology (STROBE) reporting guideline.

### Study Design and Population

This retrospective cohort study used data from the electronic medical record (EMR). We included patients 18 years or older admitted to an inpatient service between January 1, 2016, and December 31, 2019, at a single academic medical center within a large integrated health care system in New England. Eligible patients met criteria for an adult sepsis event (ASE). Data were analyzed between January 8, 2021, and March 2, 2023.

Patients with an ASE were identified using methodology outlined by the Hospital Toolkit for Adult Sepsis Surveillance produced by the Centers for Disease Control and Prevention.^23^ To meet the ASE definition, a patient must have had blood cultures obtained, have been treated with antibiotics for at least 4 qualifying days, and have had evidence of organ dysfunction.

We defined the study start date, January 1, 2016, to begin after Centers for Medicare & Medicaid Services began tracking national SEP-1 quality measures.^5^ We defined the study end date, December 31, 2019, to exclude the COVID-19 pandemic and eliminate any potential COVID-19 contributory factors. If an individual patient was admitted multiple times during this period, we selected the first hospital admission. We included only patients who received antibiotics within the first 48 hours of hospitalization to focus on those whose primary problem was sepsis, rather than those who may have developed sepsis as a later complication. Last, we included only patients who were admitted to the hospital from the emergency department to avoid selecting patients transferred from other hospitals who already received multiple days of treatment.

### Exposures and Outcomes

Data were extracted from the Epic Clarity Data Warehouse (Epic Systems Corporation) EMR using structured query language. Our main exposure was preferred language, determined via documentation in the EMR. Preferred language is self-reported during patient registration and can be edited at any point of patient contact. To validate language preference documentation, we reviewed 5% of patient records and compared language preference described in medical notes with the preferred language documented in the Epic EMR. For records where a patient’s language was not listed, we conducted a manual review to confirm language preference. The primary outcome was in-hospital mortality, defined as death during a patient’s first sepsis-related inpatient admission.

### Covariates

We hypothesized based on prior studies^8,9,10,11,24,25^ and our team’s clinical expertise that the covariates below could each be associated with sepsis mortality. We collected demographic data, including sex, age, and racial and ethnic data. Racial and ethnic data were self-reported data points in the EMR and included in the demographic data given known correlations between race and ethnicity and various health outcomes^7^ and connections between language spoken and race and ethnicity. During the study period, Hispanic or Latinx ethnicity was frequently characterized as race in the EMR. To account for this, we created a composite variable for race and ethnicity. Patients who identified as Hispanic or Latinx were categorized as such and are hereinafter referred to as Hispanic. Those who did not identify as Hispanic or Latinx were categorized into their self-reported non-Hispanic or non-Latinx race categories and are hereinafter referred to as non-Hispanic. When necessary due to sample size limitations, we collapsed the race and ethnicity variable to non-Hispanic White and racial and ethnic minority subgroups (including American Indian or Alaska Native, Asian, Black, Hispanic, Native Hawaiian or Other Pacific Islander or other race or ethnicity, and multiracial) for analyses.

We collected socioeconomic variables including educational level, insurance status, and the neighborhood Area Deprivation Index for each patient’s zip code. The Area Deprivation Index is a measure created by the Health Resources and Services Administration including factors related to income, educational level, employment, and housing quality to rank neighborhoods by socioeconomic disadvantage.^26,27^ We quantified the burden of medical comorbidities from the outpatient problem list via the Elixhauser Weighted Comorbidity Index, a method based on the *International Statistical Classification of Diseases and Related Health Problems, Tenth Revision*, diagnosis codes, using the R comorbidity package.^28^ We collected data regarding the severity of illness by need for intensive care unit–level care, mechanical ventilation, and vasopressors.^29^ Mechanical ventilation was determined by the documentation of ventilator settings in the EMR. Vasopressor use was determined by pharmaceutical class in the medication administration record.

### Statistical Analysis

To estimate power, we used an expected sepsis mortality rate of 30%, based on averages from multiple US sepsis reports.^30,31^ We used an expected difference of 10% mortality between the EP and LEP groups with an α of .05, a conservative estimate given the 30% difference in mortality found in the study conducted at University of California, San Francisco.^18^ We used a ratio of 9:1 of patients with EP:LEP based on data that 10% of patients at our medical center identified as having LEP in 2019. Aiming for a power of 0.8, we estimated needing a total of 2010 patients, including 201 in the LEP group and 1809 in the EP group to be adequately powered for our primary outcome of inpatient mortality.

#### Bivariate Analysis

We first conducted unadjusted analyses of the association between inpatient mortality and language preference. We then conducted bivariate models in which the patient’s language status (EP or LEP) was paired with each patient characteristic. We used generalized estimating equations Wald tests, clustering by admission service, to assess the associations between each characteristic and the outcome, controlling for EP and LEP status.

#### Interaction Assessment

We expected race and ethnicity to be either a confounder, given its independent association between preferred language and mortality, or an effect modifier. We first tested for interaction between race and ethnicity and language preference to determine the need for stratification, using a multivariable model for mortality that contained all possible covariates along with each of those covariates multiplied by the race and ethnicity variable as interaction terms. We then looked at the *P* value for the interaction term of LEP with race and ethnicity. If there was significant interaction between LEP and race and ethnicity, we planned for a stratified analysis using 2 separate models for the race and ethnicity combinations (non-Hispanic White and racial and ethnic minority subgroups).

#### Multivariable Analysis

In adjusted analyses, we conducted logistic regression with generalized estimating equations, clustering by admission service. We applied 2 analytic approaches: analysis of covariance (ANCOVA) and propensity score adjustment. Based on our number of covariates and expected number of deaths, the ANCOVA model was likely to be overfitted, while the propensity score adjustment allowed for more covariates to be stably modeled. We performed both analyses to confirm agreement. The ANCOVA process consisted of 1 step, where all covariates of interest were included as independent variables along with EP or LEP status. In the adjusted analysis, the Asian, Black, Hispanic, and Native Hawaiian or Other Pacific Islander groups were too small to be stably modeled separately, so these categories were grouped as racial and ethnic minority groups. Stratified analyses are thus reported for racial and ethnic minority compared with non-Hispanic White subgroups. For the second approach, we generated a propensity score for each patient by running a model with EP or LEP as the outcome and all covariates of interest as independent variables. The goal was to balance the covariates between the EP and LEP groups. Next, the propensity score was placed in the main multivariable model as an independent variable along with EP or LEP. We used weighted standardized mean differences to assess goodness of fit of the propensity-adjusted models, using a difference of less than 0.10 between EP and LEP characteristics as acceptable. In both analytic approaches, we generated odds ratios (ORs) and 95% CIs that described the association between EP or LEP and the outcome of interest. We used SAS, version 9.4 (SAS Institute Inc) for all analyses, considered 2-sided *P* < .05 to be significant, and considered less than 10% missingness to be acceptable.

## Results

Initially, 10 437 patients in the study period met Centers for Disease Control and Prevention criteria for an ASE during their first hospital admission to Brigham Women’s Hospital. After inclusion criteria were applied, we had a final sample size of 2711 patients. For 57 patients, preferred language was unavailable, and we conducted a manual record review. Of those 57 patients, 51 had EP and 4 had LEP. One patient who was nonverbal and another with no language documented were removed, resulting in a final sample of 2709, within our power calculation target range (Figure). All models controlled for clustering by admission service and used a sample size of 2702, as 7 patients were missing admission service (eTable 1 in Supplement 1).

**Figure.**
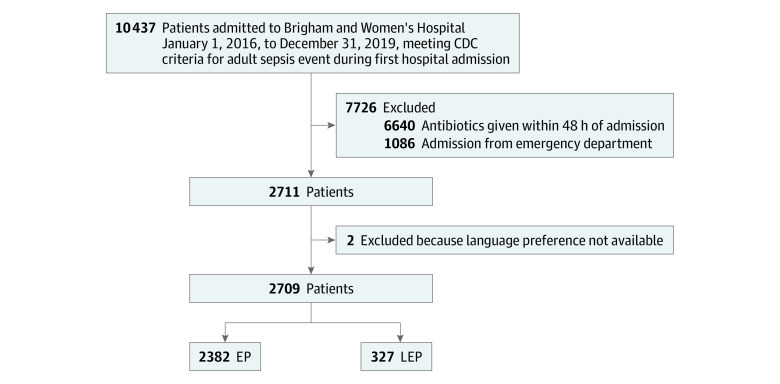
Inclusion Criteria and Study Cohort Centers for Disease Control and Prevention (CDC) definition of adult sepsis event includes (1) presumed infection, with blood culture obtained; (2) 4 qualifying antimicrobial days; and (3) organ dysfunction within the 4 qualifying antimicrobial days. EP indicates English-proficiency group; LEP, limited English proficiency group.

Two investigators (N.P.L. and H.R.) performed a manual validation of 5% of the records where language was listed. Of the 131 records screened, there were 123 in which the EMR data agreed with manual validation, and 11 of these 123 patients had LEP. For all 8 records in which there were discrepancies, the EMR data listed the patient as having EP, but the record review showed that the patient had LEP.

Patient characteristics are shown in Table 1. Educational level was not included due to missingness. In total, patients had a mean (SD) age of 65.0 (16.2) years; 1523 were men (56.2%) and 1186 (43.8%) were women. In terms of race and ethnicity, 9 patients (0.3%) were American Indian or Alaska Native, 101 (3.7%) were Asian, 314 (11.6%) were Black, 226 (8.3%) were Hispanic, 38 (1.4%) were Native Hawaiian or Other Pacific Islander or of other race or ethnicity, 1968 (72.6%) were White, and 6 (0.2%) were multiracial. Age and sex were similar between the EP and LEP groups. The LEP group had a higher percentage of Hispanic and Asian patients and patients with Medicaid coverage. Spanish was the most common language among patients in the LEP group. The most common languages among the 95 non-Hispanic White patients in the LEP group were Arabic (34 [35.8%]), Russian (24 [25.3%]), and Portuguese (13 [13.7%]). For the 228 racial and ethnic minority subgroup patients in the LEP group, the most common languages were Spanish (142 [62.3%]) and Chinese (29 [12.7%]). eTable 2 in Supplement 1 includes a further breakdown of languages spoken.

**Table 1.** Characteristics of Patients With EP and LEP^a^

Characteristics (N = 2709)	Patient group			*P* value
	Total (N = 2709)	EP (n = 2382)	LEP (N = 327)	
Age, mean (SD), y	65.0 (16.2)	64.9 (16.0)	66.3 (17.4)	.40
Sex				
Men	1523 (56.2)	1348 (56.6)	175 (53.5)	.17
Women	1186 (43.8)	1034 (43.4)	152 (46.5)	
Race and ethnicity				
American Indian or Alaska Native	9 (0.3)	9 (0.4)	0	<.001^b^
Asian	101 (3.7)	54 (2.3)	47 (14.4)	
Black	314 (11.6)	287 (12.0)	27 (8.3)	
Hispanic or Latinx	226 (8.3)	91 (3.8)	135 (41.3)	
White	1968 (72.6)	1873 (78.6)	95 (29.1)	
Other^c^	38 (1.4)	20 (0.8)	18 (5.5)	
Multiracial	6 (0.2)	5 (0.2)	1 (0.3)	
Missing	47 (1.7)	43 (1.8)	4 (1.2)	
Insurance				
Commercial	965 (35.6)	899 (37.7)	66 (20.2)	<.001
Medicaid, MassHealth, or low income	325 (12.0)	252 (10.6)	73 (22.3)	
Medicare	1318 (48.7)	1183 (49.7)	135 (41.3)	
VA, worker’s compensation, or other	58 (2.1)	21 (0.9)	37 (11.3)	
Missing	43 (1.6)	27 (1.1)	16 (4.9)	
Area Deprivation Index^d^				
Mean (SD)	20.6 (13.1)	20.8 (13.2)	19.6 (12.1)	.34
Missing	143 (5.6)	92 (3.9)	51 (15.6)	
Admission year				
2016	643 (23.7)	556 (23.3)	87 (26.6)	.48
2017	663 (24.5)	584 (24.5)	79 (24.2)	
2018	687 (25.4)	611 (25.7)	76 (23.2)	
2019	716 (26.4)	631 (26.5)	85 (26.0)	
Elixhauser Weighted Comorbidity Index score^e^				
Mean (SD)	9.2 (10.6)	9.2 (10.5)	9.6 (10.9)	.55
Missing	41 (1.5)	37 (1.6)	4 (1.2)	
Overall length of stay, mean (SD), d	13.2 (15.7)	13.1 (14.5)	14.2 (22.8)	.52
ICU transfer in first 48 h				
Yes	1280 (47.2)	1139 (47.8)	141 (43.1)	.15
No	1429 (52.8)	1243 (52.2)	186 (56.9)	
Mechanical ventilation in first 48 h				
Yes	587 (21.7)	534 (22.4)	53 (16.2)	.01
No	2122 (78.3)	1848 (77.6)	274 (83.8)	
Vasopressors in first 48 h				
Yes	1008 (37.2)	896 (37.6)	112 (34.3)	.41
No	1701 (62.8)	1486 (62.4)	215 (65.7)	

Abbreviations: EP, English proficiency; ICU, intensive care unit; LEP, limited English proficiency; VA, Veterans Affairs.

^a^ Unless otherwise indicated, data are expressed as No. (%) of patients. Percentages are rounded and may not total 100.

^b^ Calculated for dichotomous variable used in our analysis as non-Hispanic White compared with racial and ethnic minority subgroups.

^c^ Includes Native Hawaiian or Other Pacific Islander and other race or ethnicity.

^d^ Includes 2566 patients, 2290 in the EP and 276 in the LEP groups. Scores range from 1 to 100, with higher scores indicating higher levels of disadvantage.

^e^ Includes 2668 patients, 2345 in the EP and 323 in the LEP groups. Scores range from −18 to 59, with higher scores indicating a patient has a higher sum of weighted comorbidities.

In the unadjusted analyses, the absolute mortality rate for patients with EP was 19.6% (446 of 2382) compared with 21.1% (69 of 327) for patients with LEP. The odds of mortality were minimally higher for the LEP compared with the EP groups; however, this difference was not significant (OR, 1.12 [95% CI, 0.88-1.42]; *P* = .38) (Table 2).

**Table 2.** Unadjusted Association Between LEP and Inpatient Mortality

Inpatient mortality by language proficiency group	Outcome, No. (%)		OR (95% CI)	*P* value
	Deceased	Alive		
Overall (N = 2709)^a^				
EP	466 (19.6)	1916 (80.4)	1 [Reference]	NA
LEP	69 (21.1)	258 (78.9)	1.12 (0.88-1.42)	.38
**Stratified by race and ethnicity (n = 2662)** ^b^				
Non-Hispanic White subgroup (n = 1968)^c^				
EP	374 (20.0)	1499 (80.0)	1 [Reference]	NA
LEP	29 (30.5)	66 (69.5)	1.80 (1.52-2.14)	<.001
All racial and ethnic minority subgroups (n = 694)^d^				
EP	78 (16.7)	388 (83.3)	1 [Reference]	NA
LEP	38 (16.7)	190 (83.3)	0.99 (0.62-1.59)	.98
**Stratified by racial and ethnic minority subgroups**				
Black				
EP	51 (17.3)	244 (82.7)	1 [Reference]	NA
LEP	6 (19.4)	25 (80.6)	1.15 (0.49-2.67)	.75
Hispanic				
EP	13 (14.3)	78 (85.7)	1 [Reference]	NA
LEP	15 (11.1)	120 (88.9)	0.75 (0.38-1.49)	.41
American Indian or Alaska Native, Asian, Native Hawaiian or Other Pacific Islander, other race or ethnicity, and multiracial				
EP	18 (14.1)	110 (85.9)	1 [Reference]	NA
LEP	23 (18.1)	104 (81.9)	1.35 (0.81-2.27)	.25

Abbreviations: EP, English proficiency; LEP, limited English proficiency; NA, not applicable; OR, odds ratio.

^a^ Includes 2702 of 2709 (99.7%) with nonmissing admission service for OR calculation.

^b^ Includes 2662 of 2702 (98.5%) with nonmissing race and ethnicity data.

^c^ Includes 1963 of 1968 (99.7%) with nonmissing admission service for OR calculation.

^d^ Includes American Indian or Alaska Native, Asian, Black, Hispanic, Native Hawaiian or Other Pacific Islander, other race or ethnicity, and multiracial.

We found a significant interaction between LEP and race and ethnicity for the mortality outcome (OR, 2.11 [95% CI, 1.03-4.35]; *P* = .04). Therefore, all further analyses were stratified by race and ethnicity. The odds of inpatient mortality were significantly higher for non-Hispanic White patients in the LEP group compared with the EP group (OR, 1.80 [95% CI, 1.52-2.14]; *P* < .001). The difference in inpatient mortality odds between the LEP and EP groups among the racial and ethnic minority subgroup was not significant (OR, 0.99 [95% CI, 0.62-1.59]; *P* = .38) (Table 2).

For the adjusted analyses, we used propensity adjustment, which had adequate goodness of fit (see eTable 3 in Supplement 1). As Table 3 shows, among non-Hispanic White patients, the odds of inpatient mortality were significantly higher for the LEP compared with the EP groups in the propensity score–adjusted analyses (adjusted OR, 1.56 [95% CI, 1.02-2.39]; *P* = .04). Among patients in the racial and ethnic minority subgroup, there were lower odds of inpatient mortality for LEP compared with EP, but this difference was not significant (OR, 0.99 [95% CI, 0.63-1.58]; adjusted OR, 0.91 [95% CI, 0.56-1.48]) (Table 3 provides grouped analysis and eTable 4 in Supplement 1 provides additional modeling of the Black and Hispanic subgroups).

**Table 3.** Propensity-Adjusted Regression Models of Association Between LEP and Inpatient Mortality^a^

Variable	Unadjusted analyses				Adjusted analyses			
	Non-Hispanic White subgroup		Racial and ethnic minority subgroup		Non-Hispanic White subgroup		Racial and ethnic minority subgroup	
	OR (95% CI) (n = 1963)^b^	*P* value	OR (95% CI) (n = 694)	*P* value	Adjusted OR (95% CI) (n = 1839)^c^	*P* value	Adjusted OR (95% CI) (n = 640)^d^	*P* value
Language								
EP	1 [Reference]	NA	1 [Reference]	NA	1 [Reference]	NA	1 [Reference]	NA
LEP	1.76 (1.41-2.21)	<.001	0.99 (0.63-1.58)	.98	1.56 (1.02-2.39)	.04	0.91 (0.56-1.48)	.71
Propensity score	NA	NA	NA	NA	38.88 (1.50-1010.49)	.03	0.75 (0.18-3.10)	.69

Abbreviations: EP, English proficiency; LEP, limited English proficiency; NA, not applicable; OR, odds ratio.

^a^ Propensity score was created from a regression model with EP or LEP as the outcome and the following covariates: admission service, sex, age, Elixhauser Weighted Comorbidity Index score, mechanical ventilation initiated in first 48 hours, vasopressor initiated in first 48 hours, intensive care unit admission within first 48 hours, insurance payor, hospital admission year, and Area Deprivation Index.

^b^ Includes 1963 of 1968 (99.7%) with nonmissing admission service for OR calculation.

^c^ Includes 1839 of 1968 (93.4%) with nonmissing covariates for adjusted OR calculation.

^d^ Includes 640 of 694 (92.2%) with nonmissing covariates for adjusted OR calculation.

We also performed a sensitivity analysis with ANCOVA models for the inpatient mortality outcome, again clustering by admission service and controlling for all variables that were incorporated into the propensity score (eTable 5 in Supplement 1). Among non-Hispanic White patients, the odds of inpatient mortality were significantly higher for the LEP group compared with the EP group (adjusted OR, 1.80 [95% CI, 1.40-2.33]; *P* < .001). Among patients in racial and ethnic minority subgroups, the difference in odds of mortality between the LEP and EP groups was not significant (OR, 0.90 [95% CI, 0.53-1.51]; *P* = .68).

## Discussion

Patients with LEP have demonstrated worse health outcomes compared with their counterparts with EP across multiple studies.^13,14^ However, few studies have looked at the association of LEP with sepsis, one of the most common inpatient diagnoses with significant morbidity and mortality. The findings of this cohort study suggest that at a large New England urban tertiary care center, there was no overall difference in inpatient mortality among LEP patients compared with EP patients with sepsis. However, the indicated stratified analysis shows that White patients with LEP had significantly higher mortality than their counterparts in an EP group, even when adjusting for other factors. This difference was not present among other racial and ethnic categories. Our overall findings and the major potential inequity in subgroup outcomes suggest that language-based inequities exist for patients experiencing sepsis.

Overall, we expected increased odds of inpatient mortality across all LEP groups in our study. However, only the non-Hispanic White patients in the LEP group, our largest racial and ethnic subgroup, demonstrated a strong association with worse sepsis outcomes. The racial and ethnic minority subgroups with LEP, who were less represented, did not show this association. To our knowledge, these findings have not yet been demonstrated in other studies. They differ from the prior study conducted at a large medical center in California,^18^ which demonstrated that LEP was associated with higher mortality across all racial and ethnic groups except Black and Latinx. These differences likely demonstrate local contextual variation and other intersectional drivers of unequal health outcomes. They highlight the need for focused research to identify language-based inequities and their drivers across various contexts.

Within our study’s local context, our findings have multiple potential explanations. First, there may be differences in the access to and modality of interpreter services for different LEP subgroups. The most common language spoken by patients with LEP overall in this study was Spanish, but in the non-Hispanic White subgroup, the most common languages were Arabic and Russian. Interpreter services at our hospital are provided by certified interpreters, but the time to reach an interpreter and modality of interpretation (in person, by video, or by telephone) can vary by language. Multiple studies^32,33^ have shown that an in-person interpretation provides a higher-quality service, and high-quality interpretation leads to improved patient outcomes. We do not have specific data on interpreter service use for each encounter, but delays and use of telephone-based interpretation may occur more frequently for less common languages like Arabic and Russian. These delays and lower quality of interpreting services could make it more difficult for patients to express symptoms, leading to missed early warning signs of sepsis and the potential patient safety events shown to have a higher incidence in populations with LEP.^34^ Granular information was not available for this study regarding interpreter services or safety events, but future studies should investigate interpreter service access, use, and quality, along with incidence of patient safety events to better understand contributory factors.

Bias could also play a role in the disparity in sepsis outcomes for White patients with LEP compared with their White counterparts with EP. Clinicians’ implicit biases could factor into deciding when to call an interpreter. Resident physicians have been found to underuse interpreters in general, citing time constraints and other barriers.^35^ It is possible that White patients with LEP were presumed to be more comfortable in English than patients with LEP in racial and ethnic minority subgroups based on appearance, leading to underuse of interpreters for White patients with LEP and worse outcomes. Potential institutional biases could also have played a role. Several studies at our own institution have shown significant disparities in triage, care management, and security calls based on race, ethnicity, and language.^36,37,38^ Potential inequities in care processes for sepsis between patients with LEP and EP as well must be further elucidated.

At Brigham and Women’s Hospital, where this study took place, we have started addressing these inequities through departmental initiatives such as a video interpreter pilot conducted by several of this study’s authors (N.P.L, W.R.M., H.R., A.V., and E.G.) to identify discrimination and bias in hospital safety events. As our cohort study has noted, patients’ language preferences must be respected and integrated into their medical care, regardless of race or ethnicity. We must improve care for patients with LEP and other patients experiencing inequities that still exist nearly 20 years after the Institute of Medicine established the pillar of equity in care.

### Limitations

There are several limitations to this study. First, it has limited generalizability, as this population is derived from a single urban tertiary academic teaching hospital. Second, there were inconsistencies in preferred language documentation and underreporting of LEP in the EMR, which would result in misclassification bias, where patients with LEP are erroneously classified as having EP. This could attenuate the association between LEP and sepsis mortality. Third, while we included only patients admitted to the hospital through the emergency department, a subset of patients transferred from other hospitals are admitted through the emergency department. This means some of the patients in our sample may have received initial care at another hospital, which could have affected their outcomes. Last, though we have included multiple covariates, there may be additional factors we did not include that affect sepsis outcomes.

## Conclusions

The findings of this cohort study suggest that with such a large disparity in sepsis outcomes for White patients with LEP compared with White patients with EP, further research is needed to examine factors perpetuating this inequity, whether they may be interpersonal, institutional, or structural in nature. Sepsis is a common discharge diagnosis among many medical institutions and affects a demographically heterogenous group of people. The inequities found here may exist across other diagnoses as well. Most importantly, these findings call for targeted interventions to promote language equity, as the Institute of Medicine initially called for in 2004.

## Notes

Supplement 1. **eTable 1.** Patients per Admission Service **eTable 2.** Language Distribution Among Patients With EP and LEP **eTable 3.** Weighted Standardized Mean Differences in Propensity-Adjusted Models Estimating Inpatient Mortality **eTable 4.** Propensity-Adjusted Models of Association Between LEP and Inpatient Mortality for Black and Hispanic Subgroups **eTable 5.** ANCOVA Models of Association between LEP and Inpatient Mortality

Supplement 2.
Data Sharing Statement
