# Supplementary material for: Limited English Proficiency and Sepsis Mortality by Race and Ethnicity
Source: JAMA Netw Open. 2024 Jan 4;7(1):e2350373. doi: 10.1001/jamanetworkopen.2023.50373 (PMC10767592; doi:10.1001/jamanetworkopen.2023.50373)
Supplement: Supplement 1. — eTable 1. Patients per Admission Service eTable 2. Language Distribution Among Patients With EP and LEP eTable 3. Weighted Standardized Mean Differences in Propensity-Adjusted Models Estimating Inpatient Mortality eTable 4. Propensity-Adjusted Models of Association Between LEP and Inpatient Mortality for Black and Hispanic Subgroups eTable 5. ANCOVA Models of Association between LEP and Inpatient Mortality [file jamanetwopen-e2350373-s001.pdf]

Supplementary Online Content

Limaye NP, Matias WR, Rozansky H, et al. Limited English proficiency and sepsis mortality by race and ethnicity. *JAMA Netw Open*. 2024;6(1):e2350373. doi:10.1001/jamanetworkopen.2023.50373

eTable 1. Patients per Admission Service
eTable 2. Language Distribution Among Patients With EP and LEP
eTable 3. Weighted Standardized Mean Differences in Propensity-Adjusted Models Estimating Inpatient Mortality
eTable 4. Propensity-Adjusted Models of Association Between LEP and Inpatient Mortality for Black and Hispanic Subgroups
eTable 5. ANCOVA Models of Association between LEP and Inpatient Mortality

This supplementary material has been provided by the authors to give readers additional information about their work.

© 2024 Limayer NP et al. *JAMA Network Open*.

eTable 1. Patients per Admission Service

Admission Service	Frequency	Percent	Cumulative Frequency	Cumulative Percent
**Missing**	7	0.26	7	0.26
**Bone marrow transplant/Transplant surgery**	40	1.48	47	1.73
**Cardiac/Vascular Surgery**	27	1.00	74	2.73
**Cardiology**	199	7.35	273	10.08
**GGI Surgery**	25	0.92	298	11.00
**OB/GYN**	11	0.41	309	11.41
**Heme/Onc**	620	22.89	929	34.29
**Medicine/ORL***	704	25.99	1633	60.28
**Nephrology**	26	0.96	1659	61.24
**Neurology**	72	2.66	1731	63.90
**Neurosurgery**	22	0.81	1753	64.71
**Orthopedics**	19	0.70	1772	65.41
**Pulmonary medicine**	680	25.10	2452	90.51
**Thoracic surgery**	41	1.51	2493	92.03
**Urology**	40	1.48	2533	93.50
**Trauma and acute surgery/Burn/ Plastic Surgery**	176	6.50	2709	100.00

*Only 1 ORL patient in dataset, grouped with 703 medicine patients

© 2024 Limayer NP et al. *JAMA Network Open*.

eTable 2. Language Distribution Among Patients With EP and LEP

Language	Overall N = 2709	White, non-Hispanic/ Latinx (N = 1968)	Non-White (N = 694)	Missing (n=47)
**English proficient**	2382 (87.9)	1873 (95.2)	466 (67.2)	43 (91.5)
**Limited English proficient**	327 (12.1)	95 (4.8)	228 (32.9)	4 (8.5)
**Spanish**	145 (44.3)	3 (3.2)	142 (62.3)	0
**Arabic**	46 (14.1)	34 (35.8)	10 (4.4)	2 (50.0)
**Chinese**[#]	30 (9.2)	1 (1.1)	29 (12.7)	0
**Russian**	24 (7.3)	24 (25.3)	0	0
**Portuguese**	20 (6.1)	13.(13.7)	6 (2.6)	1 (25.0)
**Haitian-Creole**	19 (5.8)	0	19 (8.3)	0
**Other**	43 (13.2)	20 (21.1)	22 (9.7)	1 (25.0)
**Missing**	0	0	0	0

*Other languages include: Greek, Vietnamese, Albanian, Italian, French, Khmer, Hindi, Thai, and Farsi
[#]Includes all dialects (Mandarin, Cantonese, etc.)

© 2024 Limayer NP et al. *JAMA Network Open*.

eTable 3. Weighted Standardized Mean Differences in Propensity-Adjusted Models Estimating Inpatient Mortality
(LEP = treated, EP = control)

Characteristics	White, non-Hispanic/Latinx model N=1839[š]	Non-white model N=640[Ÿ]
Age (years)	0.021	-0.024
Gender	0.007	0.019
Insurance		
Commercial	-0.011	0.013
Medicaid/MassHealth/Low income	0.091	0.002
Medicare	-0.034	-0.005
VA/Worker’s comp/Other	-0.049	-0.030
Area deprivation index	-0.020	0.032
Admission year		
2016	0.013	0.001
2017	-0.001	0.012
2018	-0.012	-0.018
2019	-0.001	0.004
Elixhauser score	0.003	0.017
ICU transfer in first 48 hours	-0.008	0.014
Mechanical ventilation in first 48 hours	0.017	0.026
Vasopressors in first 48 hours	-0.002	0.027

[š]N = 1839/1968 (93.45%) with non-missing covariates
[Ÿ]N= 640/694 (92.22%) with non-missing covariates

© 2024 Limayer NP et al. *JAMA Network Open*.

eTable 4. Propensity-Adjusted* Models of Association Between LEP and Inpatient Mortality for Black and Hispanic Subgroups

Predictor	Black Adjusted OR (95% CI)<br><br>N= 308[Ÿ]	P-value	Hispanic/Latinx Adjusted OR (95% CI)<br><br>N=214[š]	P-value
**Language**				
EP	Referent		Referent	
LEP	1.65 (0.63-4.31)	0.31	0.66 (0.26-1.69)	0.39
**Propensity Score**	**0.06 (0.01-0.62)**	**0.02**	3.44 (0.61-19.41)	0.16

*Propensity score was created from a regression model with EP/LEP as the outcome and the following covariates: admission service, sex, age, Elixhauser score, mechanical ventilation initiated in first 48 hours, vasopressor initiated in first 48 hours, ICU admission within first 48 hours, insurance payor, hospital admission year, area deprivation index.

[Ÿ]N= 308/326 (94.48%) with non-missing covariates for aOR calculation

[š]N= 214/226 (94.69%) with non-missing covariates for aOR calculation

© 2024 Limayer NP et al. *JAMA Network Open*.

eTable 5. ANCOVA Models of Association between LEP and Inpatient Mortality*

Predictor	White, non-Hispanic/Latinx Adjusted OR (95% CI) N=1839[š]	Non-White Adjusted OR (95% CI) N=640[Ÿ]
**Language**		
English	referent	referent
LEP	**1.80 (1.40-2.33), P <0.0001**	0.90 (0.53, 1.51), P = 0.68

*All ANCOVA regression models with EP/LEP as the outcome were clustered by admission service and had the following covariates: sex, age, Elixhauser score, mechanical ventilation initiated in first 48 hours, vasopressor initiated in first 48 hours, ICU admission within first 48 hours, insurance payor, hospital admission year, area deprivation index.
[š]N = 1839/1968 (93.45%) with non-missing covariates
[Ÿ]N= 640/694 (92.22%) with non-missing covariates

© 2024 Limayer NP et al. *JAMA Network Open*.
