# Supplementary material for: Limited English Proficiency and Sepsis Mortality by Race and Ethnicity
Source: JAMA Netw Open. 2024 Jan 4;7(1):e2350373. doi: 10.1001/jamanetworkopen.2023.50373 (PMC10767592; doi:10.1001/jamanetworkopen.2023.50373)
Supplement: Supplement 2. — Data Sharing Statement [file jamanetwopen-e2350373-s002.pdf]

Data Sharing Statement

Limaye. Limited English Proficiency and Sepsis Mortality by Race and Ethnicity. *JAMA Netw Open*. Published January 04, 2024. doi:10.1001/jamanetworkopen.2023.50373

Data
Data available: Yes
Data types: Deidentified participant data, Data dictionary
How to access data: Deidentified participant data and data dictionary will be made available on request via email to one of our authors: neha.limaye@mountsinai.org, egershanik@mgh.harvard.edu, or bneville@bwh.harvard.edu
When available: With publication

Supporting Documents
Document types: None

Additional Information
Who can access the data: The de-identified participant data and data dictionary may be made available to researchers/students requesting data after the author team has approved the request.
Types of analyses: Research purposes
Mechanisms of data availability: After approval by research team.
